# The Reorientation of T-Cell Polarity and Inhibition of Immunological Synapse Formation by CD46 Involves Its Recruitment to Lipid Rafts

**DOI:** 10.1155/2011/521863

**Published:** 2011-01-20

**Authors:** Mandy J. Ludford-Menting, Blessing Crimeen-Irwin, Jane Oliaro, Anupama Pasam, David Williamson, Natalie Pedersen, Patricia Guillaumot, Dale Christansen, Serge Manie, Katharina Gaus, Sarah M. Russell

**Affiliations:** ^1^Peter MacCallum Cancer Centre, East Melbourne, VIC 3002, Australia; ^2^Department of Surgery, University of Melbourne, Parkville, VIC 3010, Australia; ^3^Centre for Vascular Research, University of New South Wales, Sydney, NSW 2052, Australia; ^4^Department of Haematology, The Prince of Wales Hospital, Sydney, NSW 2052, Australia; ^5^Laboratoire de Génétique, Signalisation et Cancer. CNRS, UMR 5201, Université Claude Bernard Lyon 1, Lyon, France; ^6^Department of Pathology, University of Melbourne, Parkville, VIC 3010, Australia; ^7^Centre for Micro-Photonics, Swinburne University of Technology, Hawthorn, VIC 3122, Australia

## Abstract

Many infectious agents utilize CD46 for infection of human cells, and therapeutic applications of CD46-binding viruses are now being explored. Besides mediating internalization to enable infection, binding to CD46 can directly alter immune function. In particular, ligation of CD46 by antibodies or by measles virus can prevent activation of T cells by altering T-cell polarity and consequently preventing the formation of an immunological synapse. Here, we define a mechanism by which CD46 reorients T-cell polarity to prevent T-cell receptor signaling in response to antigen presentation. We show that CD46 associates with lipid rafts upon ligation, and that this reduces recruitment of both lipid rafts and the microtubule organizing centre to the site of receptor cross-linking. These data combined indicate that polarization of T cells towards the site of CD46 ligation prevents formation of an immunological synapse, and this is associated with the ability of CD46 to recruit lipid rafts away from the site of TCR ligation.

## 1. Introduction

CD46 is a human receptor for complement and many pathogens, including Neisseria, Group A Streptococcus, Species B adenoviruses, vaccine strains of the measles virus, and Human Herpes Virus 6 (HHV6) [1–3]. The broad range of pathogens to which CD46 can bind, combined with the ubiquitous expression of CD46, have prompted much interest in the utility of CD46-binding viruses as oncolytic agents [4–11], for gene therapy [12–15], and as vectors for vaccination [16]. However, it is increasingly clear that CD46 not only mediates entry of these infectious agents, but also transmits signals upon ligation that can have important effects on immune responses [17–19]. Many of the pathogens that utilize CD46 as a receptor alter immune function in the host, by both direct and indirect mechanisms [1, 2, 20, 21]. Immune modulation by CD46 signaling is best studied in response to measles vaccine strains, where ligation of CD46 inhibits T cell activation and induces regulatory T cells [22–24]. The mechanisms for this process have been difficult to elucidate, in part because of the difficulty of discriminating pleiotropic effects of the pathogen from direct effects of CD46 signaling. 

However, recent work has identified cellular processes that are directly affected by CD46 ligation, and that provide an opportunity to dissect the molecular interactions through which CD46 exerts its effects. Mounting evidence suggests that CD46 signaling affects cell morphology and polarity [25, 26], and that CD46 function is regulated by intracellular compartmentalization [27, 28]. Indeed, ligation of CD46 induces polarization of the T cell towards the ligation site, subsequently preventing the formation of an immunological synapse, and reducing T cell signaling [25]. These observations indicate that alterations in cell polarity mediated by ligand binding to CD46 might impact upon multiple cellular functions and on immunological responses. Here, we establish a tractable in vitro system with which to elucidate the mechanisms by which CD46 controls polarity, and demonstrate that the changes in polarity of T cells involve a functional interaction of CD46 with lipid rafts.

## 2. Materials and Methods

### 2.1. Constructs, Cell Lines, and Reagents

**Figure 1:**
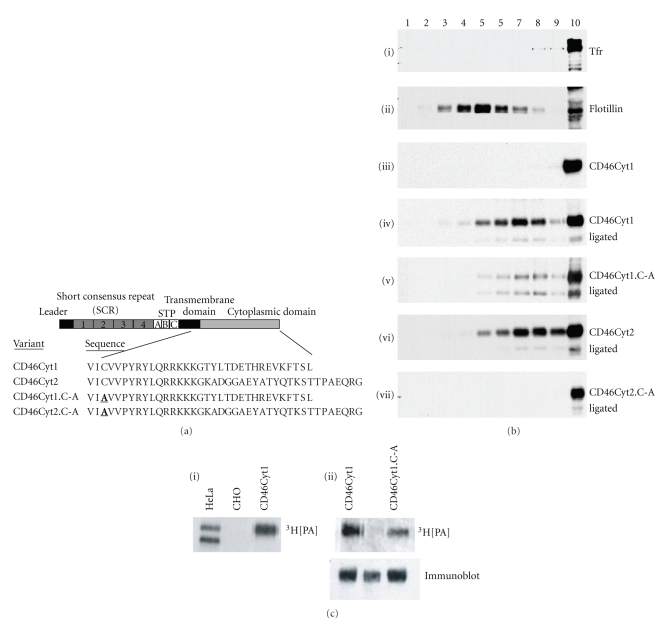
Palmitoylation regulates CD46 recruitment to DRM. (a) Schematic of CD46, with cytoplasmic sequences for the Cyt1 and Cyt2 alternatively spliced isoforms. Sequence begins within the transmembrane domain at residue 326 [28] and ends at the C-terminus. The transmembrane cysteine that we explore here is in bold and underlined. (b) MD45 cells expressing CD46 variants were either untreated (i)–(iii) or ligated with a CD46-specific antibody (iv)–(vii), lysed with TTX-100, fractionated on sucrose gradient, electrophoresed and probed with antibodies specific for Tfr, Flotillin, or CD46. Fractions 3–8 contain DRM proteins, and Fraction 10 contains the detergent-soluble protein. The lower, faint band visible in panels (iv)–(vii) represents the cross-linking antibody. (c) Cells were incubated with radiolabeled palmitoic acid, lysed and immunoprecipitated with antibodies to CD46, electrophoresed, and autoradiographed. Duplicate gels (lower panels) were immunoblotted with antibodies to CD46 to assess CD46 expression levels. (i) HeLa cells, CHO cells, and CHO cells transfected with CD46Cyt1. (ii) MD45 cells transduced with CD46Cyt1 or CD46Cyt1.C-A as shown. These data are representative of 3 independent experiments.

CD46Cyt1.C-A and CD46Cyt1.C-A,L-R were generated by site-directed mutagenesis as described [27], and with CD46-Cyt1, CD46-Cyt2, and CD46-Cyt1L-R [28] expressed in the CHO-K1 cell line [29], and subcloned into pMSCV-GFP for expression in the MD45 cell line [27]. Expression was at approximately endogenous levels (see comparison with HeLa cells in Figure 1(c)). Human T cells were isolated as described [27]. Antibodies were mouse IgG1 to Transferrin receptor (Tfr, CD71) (BD Pharmingen, San Diego, CA); mouse IgG1 to flotillin-2 (BD Transduction Laboratories Franklin Lakes, NJ), mouse IgG2a (E4.3) and polyclonal rabbit (1840) to CD46 [29, 30].

### 2.2. Cell Surface Ligation and Immunofluorescent Staining

MD45 cells (4 × 10^4^) expressing CD46 constructs were ligated with anti-CD46 (1840 rabbit polyclonal) antibody or anti-Tfr antibody for 30 min at 37°C, then incubated with antiCD3/CD28 coated beads and stained as described [25]. Confocal images were acquired and processed with a BX61 microscope (Olympus, Melville, NY) and Olympus Fluorview FV1000 laser-scanning confocal and software (Olympus, Japan) as described [27]. 3D images of the cells were acquired with an optical distance of 0.5 *μ*M between slices. Approximately 20–30 slices were acquired per image, with the pin hole set to 1 AU, using a 60 × oil immersion objective (NA 1.42). Digital images were processed with Image J (NIH, MD) and MetaMorph (Universal Imaging Corporation, PA). For MTOC recruitment, slides were de-identified and scored for concentration of tubulin staining at the bead interface.

### 2.3. Laurdan Microscopy

Cells labeled with Laurdan (Molecular Probes, Eugene, OR) and ligated and stimulated as above were excited with a 2-photon laser at 800 nm and emission intensities simultaneously recorded in the range of 400–460 nm and 470–530 nm [31, 32]. Intensity images were converted into Generalized Polarization (GP) images (WiT software) with

(1) $$\text{GP}=\frac{I_{\left(400\text{–}460\right)}-I_{\left(470\text{–}530\right)}}{I_{\left(400\text{–}460\right)}+I_{\left(470\text{–}530\right)}}.$$

Final GP images were pseudocolored in Adobe Photoshop. For all images a 100 × oil objective, *N*_*A*_ = 1.4 was used. GP histograms were fitted to two Gaussian populations using the nonlinear fitting algorithm Solver in Microsoft Excel [31]. The mean GP value of membranes adjacent to the bead or of pixels masked by CD46 staining was calculated as described [31, 33]. Statistical analysis was performed by ANOVA with Tukey's multiple comparison tests.

### 2.4. Isolation of Detergent Resistant Membranes (DRM)

Cells were scraped, lysed at 3 mg proteins/ml in ice-cold TNE buffer (TBS pH 7.2, 4 mM NaVO_4_, 5 mM EGTA and protease inhibitors) containing 0.5% Triton X-100 (Sigma) for 25 min at 4°C and fractionated on a bottom-loaded discontinuous sucrose gradient as described [34]. Gradient fractions were TCA precipitated and analyzed by Western blot. For ligation, cells were incubated with anti-CD46 (1840, 20 min, 5 *μ*g/mL, 4°C) [29] and with secondary antibody (5 min, 10 *μ*g/mL, 37°C).

### 2.5. Palmitoylation Assay

Cells (1 × 10^6^) were cultured with 5 mCi (total) ^3^H palmitoic acid (Amersham Biosciences UK) for 4 hours at 37°C and 5% CO_2_, washed once in PBS, lysed in 0.5% Nonidet-P40, TBS (pH7.8), 5 mM EDTA, and Complete protease inhibitors (Roche Diagnostics, Australia) on ice, and immunoprecipitated with anti-CD46 (1840) antibody for PAGE.

## 3. Results

### 3.1. CD46 Is Recruited into Detergent-Resistant Membranes by Ligation, via a Palmitoylated Cysteine in the Transmembrane Domain

Lipid rafts, defined as “small (10–200 nm), heterogeneous, highly dynamic, sterol- and sphingolipid-enriched domains that compartmentalize cellular processes” [35], have been implicated in T-cell polarity [36–40], so we proposed that they might mediate CD46 effects on polarity. In support of the notion that CD46 might associate with rafts, a recent study showed that CD46 was recruited to detergent-resistant membranes (DRM) after infection with Human Herpes Virus 6 (HHV6) [41]. CD46 is alternatively spliced to yield two isoforms with different cytoplasmic domains (CD46Cyt1 and CD46Cyt2, Figure 1(a)). Each isoform contains a transmembrane cysteine that is a possible site for palmitoylation, which might enable recruitment into lipid rafts [30, 42]. Multiple CD46 isoforms are expressed in every human cell, so to determine the palmitoylation and raft recruitment of individual isoforms, we utilized the murine T-cell line, MD45 [27], which does not normally express CD46 [1, 30]. First, MD45 cells expressing CD46Cyt1 were left untreated or ligated with antibody to CD46, lysed, and fractionated on a sucrose density gradient (Figure 1(b)). The lipid raft marker flotillin (ii), but not the nonraft protein transferrin receptor (Tfr) (i), was present in the DRM (fractions 3–8). No CD46 was detected in the DRM from nonligated cells (iii); however, a portion of the wild-type CD46 was recruited into DRM after ligation (iv). These data add further support to the notion that CD46 is recruited into lipid rafts upon ligation [41], providing a possible mechanism for the CD46-induced polarity changes. However, potential artefacts associated with detergent extraction [36] mean that alternative means of assessing raft association are required to confirm this hypothesis.

To test whether the transmembrane cysteine residue of CD46 provides a site for palmitoylation, cells were incubated with radiolabeled palmitoic acid, and immunoprecipitated for CD46. Radiolabeled bands of appropriate size for CD46 were detected from HeLa cells expressing endogenous CD46, and from CHO cells transfected with CD46Cyt1, but not from nontransfected CHO cells, indicating that CD46 is constitutively palmitoylated (Figure 1(c)(i)). In transfected MD45 cells, CD46Cyt1 was also palmitoylated, but mutation of the cysteine at position 328 (CD46Cyt1.C-A) completely abolished palmitoylation (Figure 1(c)(ii)). Similarly, CD46Cyt2 was palmitoylated, but only when the transmembrane cysteine was present (not shown). Similar to CD46Cyt1, CD46Cyt2 was recruited to DRM (Figure 1(b)(vi)), again dependent upon the cysteine residue (Figure 1(b)(vii)). These data combined indicate that both CD46 isoforms are recruited to DRM upon ligation, and that optimal recruitment requires palmitoylation of the transmembrane cysteine.

### 3.2. CD46 Is Recruited to Ordered Membranes upon Ligation

**Figure 2:**
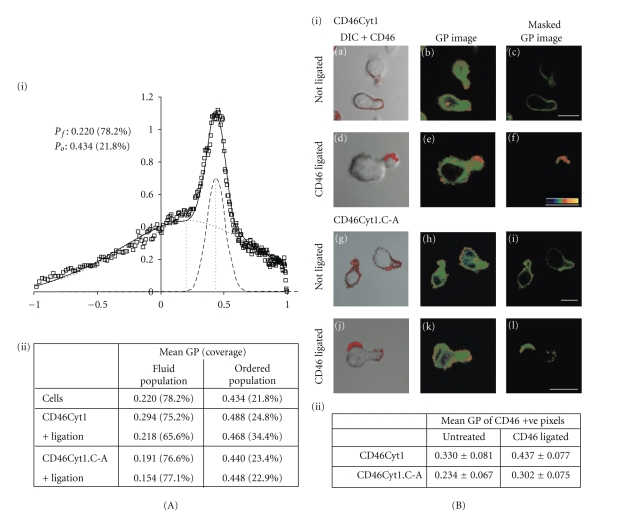
CD46 alters membrane organization and is recruited to ordered membranes. (A) The GP histograms (open squares) of ~50 cells for each cell type (untransfected cells shown, (A)(i)) were normalized and the distribution fitted onto two Gaussian populations: a fluid population P_f_ (dotted line) and a raft-like ordered population P_o_ (dashed line). The table (A)(ii) summarizes a similar analysis of GP histograms in cells transfected as shown, with or without CD46 ligation. The table gives the mean GP value that was derived by fitting two populations as per the graph above. Also shown, in brackets, is the proportion of membranes associated with either of the two populations (termed coverage). (B)(i) Laurdan-labeled MD45 cells expressing CD46Cyt1 (a)–(f) or CD46Cyt1.C-A (g)–(l) were left untreated (a)–(c) and (g)–(i) or incubated with CD46-ligating antibodies (d)–(f) and (j)–(l) before fixation and imaging. Left column shows transmission images with overlaid CD46 confocal staining in red. The middle column shows pseudocolored GP images of the identical focal depth as the CD46 confocal images. GP color scale is indicated in (f) with GP values ranging from −1 (blue) to +1 (yellow). The right column shows the masked image, in which the GP values are shown only for those pixels that were defined as positive for CD46 (red coloring in (a), (d), (g), and (j)). Table (B)(ii) summarizes the GP mean ± SD of CD46-positive membranes of 40–50 images. GP values with and without ligation are significantly different (*P* < .05) for both CD46Cyt1 and CD46Cyt1.C-A, and CD46Cyt1 is significantly different to CD46Cyt1.C-A (*P* < .05), both with and without ligation. The data is representative of 3 independent experiments.

Detergent extraction alone is not always a reliable indicator of raft recruitment [36, 39, 40], so to test whether the results above represented *bona fide* association with lipid rafts, we utilized a complementary approach based upon the analysis of ordered membrane structure using 2-photon microscopy. The fluorescent probe, Laurdan, integrates into all membranes but demonstrates a different emission profile if localized to the ordered domains that are considered to represent lipid rafts [31, 43, 44]. We ascribe a normalized ratio of the two emission regions (General Polarisation or GP) as a relative measure of membrane order, where fluid domains are arbitrarily defined as approximately 0.05–0.25, and ordered domains are approximately 0.25–0.55 [31]. Firstly, we tested whether expression and ligation of CD46 altered the physical properties of the plasma membrane globally (Figure 2(A)). In normalized GP histograms (Figure 2(A)(i)), we identified two populations of membranes that are characterized by the mean GP value (centre of the population) and its abundance (area under the curve). Hence, an ordered membrane population with high mean GP, “P_o_”, was discriminated from a broader, fluid population, “P_f_” [33]. Note that the mean GP here does not reflect an arbitrary “cut-off” such as the 0.25 above but accommodates the broad range of GP values within the population defined by curve-fitting. Comparison of these two populations under different conditions (Figure 2(A)(ii)) indicated that the proportion of condensed membranes as a percentage of total membranes was 21.8% (standard deviation 2.7%) in parental cells, increased slightly but not significantly (to 24.8%) upon transfection of CD46Cyt1, and further increased (to 34.4%, *P* < .05) upon ligation of CD46Cyt1. In contrast, transfection and ligation of CD46Cyt1.C-A had no significant effect on global membrane order (23.4% and 22.9% with or without ligation, *P* < .5 compared to nontransfected cells). These data indicate that expression of CD46 influences the organisation of the membrane fluidity, and that ligation of CD46 further enhanced the formation of ordered domains. Notably, CD46-mediated membrane condensation required the transmembrane cysteine. 

We next determined whether CD46 was preferentially recruited into ordered domains. To correlate CD46 with ordered domains, we used positive CD46 staining to mask the GP images (compare total GP image in column 2 with masked image in column 3, Figure 2(B)(i)). A high GP value in a CD46-positive pixel gives an indication of CD46 localisation in ordered domains that biophysically resemble lipid rafts. The average GP value of CD46Cyt1 positive pixels over multiple cells was 0.330 ± 0.081 (Figure 2(B)(ii), compared with an average of 0.234 ± 0.067 for CD46Cyt1.C-A (*P* < .05), indicating that palmitoylation of CD46 indeed targets the protein to more ordered domains. Ligated CD46Cyt1 was recruited to regions of average GP values 0.437 ± 0.077 (*P* < .001 compared with unligated CD46Cyt1, predominantly red colouring in Figure 2(B)(i)(d) indicating that CD46Cyt1 was recruited to highly ordered raft domains after ligation. Ligation of CD46Cyt1.C-A increased the mean GP of CD46 positive pixels (*P* < .05 for untreated compared with CD46 ligated), but not to the levels indicative of ordered domains (average GP 0.302 ± 0.075, predominately green colouring in Figure 2(B)(i)(l), *P* < .05 for CD46-ligated CD46Cyt1 compared with CD46-ligated CD46Cyt1.C-A). These studies together demonstrate that (i) CD46 constitutively localizes to a domain that, although not a classic raft as defined by detergent insolubility or GP values, is somewhat more ordered than the general cell membranes, (ii) CD46 is recruited to stable lipid rafts upon ligation, thus increasing the abundance of ordered domains, and (iii) recruitment of CD46 into ordered domains (both constitutive and ligated) is dependent upon a transmembrane cysteine.

### 3.3. CD46 Ligation Reduces Raft Accumulation at the Site of TCR Activation

**Figure 3:**
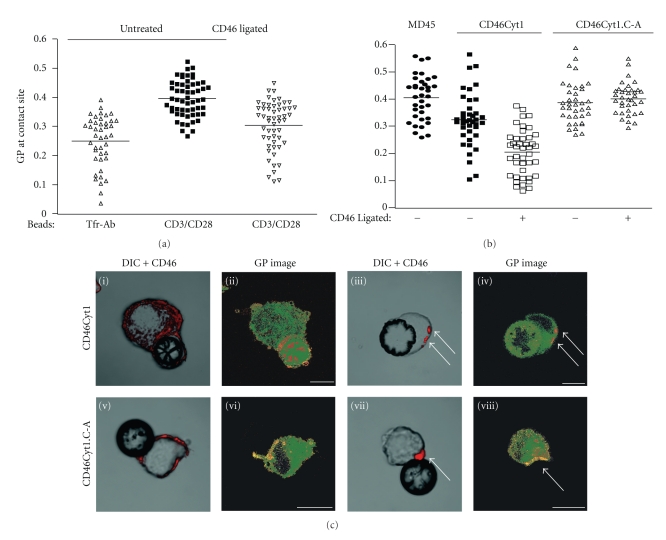
CD46 ligation reduces raft accumulation at the immunological synapse, dependent upon the transmembrane cysteine. (a) and (b), Laurdan-labeled T cells (CD8+ human T cells (a), or CD46Cyt1 transfected MD45 cells (b)) were conjugated to beads coated with antibodies against Tfr or CD3/CD28 as indicated and activated at 37°C for 7 min, fixed and imaged. Where indicated, CD46 was first ligated for 30 min. The GP value of the membranes adjacent to the beads was measured. Each symbol represents one activation site; mean values for each condition are indicated by horizontal bars. Representative images from the CD46 transduced populations in (b) are shown in (c). Laurdan-labeled MD45 cells expressing either CD46Cyt1 (i)–(iv) or CD46Cyt1.C-A (v)–(viii) were untreated ((i), (ii), (v), (vi)) or incubated with CD46-ligating antibodies ((iii), (iv), (vii), (viii)), fixed and imaged. Panels (i), (iii), (v), and (vii) show transmission images with overlaid CD46 staining in red. Panels (ii), (iv), (vi), and (viii) show GP images with the same pseudo-coloring as in Figure 2. The GP values of the membranes adjacent to the beads in the cells shown are 0.273 (ii), 0.181 (iv), 0.374 (vi), and 0.432 (viii). Arrows in (iv) and (viii) show membrane domains that contain CD46Cyt1 and CD46Cyt1.C-A, respectively. The data is representative of 3 experiments. GP values at the site of TCR stimulation were significantly (*P* < .05) different between CD46-ligated and not on the TCR-stimulated primary T cells (a), and on the CD46Cyt1-transduced MD45 cells (b).

Raft-like domains are concentrated at the immunological synapse during antigen presentation [33, 45–47]. In particular, TCR signaling components are associated with DRM [45], and the Laurdan reporter dye reveals condensed plasma membrane domains at T-cell activation sites [33]. We previously showed that ligation of CD46 abrogates recruitment of CD3 and the microtubule organizing centre (MTOC) to the site of TCR triggering [25, 48]. Because ligation of CD46 induced its association with ordered domains, we tested whether CD46 ligation alters membrane condensation at T-cell activation sites (Figure 3(a)). Activation of CD8^+^ human T cells with beads coated with antibodies to TCR components, “CD3/CD28 beads,” triggered raft formation at the cell-bead contact (GP value of 0.397 ± 0.062, compare with 0.252 ± 0.087 with negative control beads coated with antibody to Tfr, *P* < .05). Ligation of Tfr prior to stimulation with CD3/CD28 beads did not reduce membrane condensation at CD3/CD28 contact sites (unpublished data, *P* < .05). In contrast, ligation of CD46 prior to TCR stimulation significantly reduced raft formation at T cell activation sites to 0.305 ± 0.84 (*P* < .001). Thus, as well as inhibiting recruitment of MTOC and cytotoxic granules to the immunological synapse [25], ligation of CD46 prevents lipid raft recruitment to the site of TCR stimulation. 

Secondly, we tested whether association of CD46 with rafts was required for the inhibitory effect on raft recruitment to the IS, utilizing MD45 cells (Figures 3(b) and 3(c)). As in the human T cells, ordered domains were enriched at the site of interaction with CD3/CD28 beads (0.406 ± 0.082), but expression of CD46Cyt1 reduced (0.326 ± 0.098, *P* < .05), and ligation abrogated (0.207 ± 0.084) this recruitment. However, expression (0.388 ± 0.077) and ligation (0.403 ± 0.057) of CD46Cyt1.C-A had no effect. Ligated CD46Cyt1 colocalized with ordered domains (Figure 3(c), compare arrows in (iii) and (iv)), and ligated CD46Cyt1.C-A colocalized with more fluid domains (compare arrows in (vii) and (viii)). We observed a small but significant (*P* < .05) reduction in raft recruitment to CD3/CD28 beads when CD46Cyt1 was expressed and not ligated, compared to parental cells that do not express CD46 or cells expressing the CD46 palmitoylation mutant. This is consistent with the effect of CD46Cyt1 expression on global membrane organization and the location of CD46Cyt1 in ordered domains, suggesting that CD46-mediated raft domains are a separate entity to pre-existing or TCR-induced raft domains. These data together indicate that CD46 must associate with lipid rafts to prevent the polarization of rafts to the IS and that CD46 ligation competes with TCR signalling to control the localisation of higher-ordered membranes.

### 3.4. An Association with Lipid Rafts Is Required, But Not Sufficient, for the Reorientation of Polarity by CD46 Ligation

**Figure 4:**
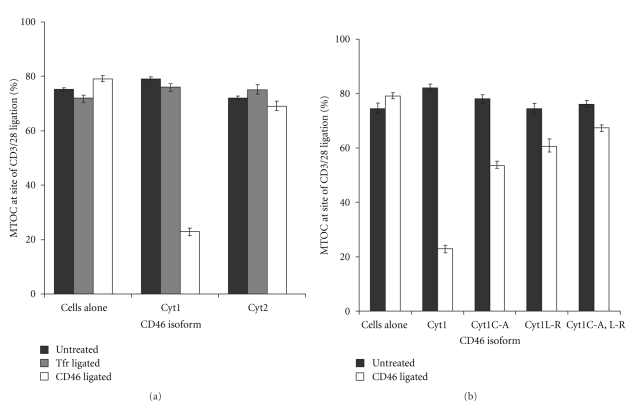
Lipid raft association is necessary but not sufficient for the control of MTOC polarization by CD46 ligation. MD45 cells, untransfected or transfected with CD46 variants as shown, were untreated (black bars) or ligated with antibodies to Tfr ((a), grey bars) or CD46 ((a) and (b), white bars), then incubated with CD3/28 beads for 90 min. Cell-bead conjugates were adhered onto glass slides by centrifugation, stained for *α*-tubulin, and examined by immunofluorescence microscopy. Samples were blind scored for the MTOC location, given as the percentage of cells in which MTOC was found at the cell-bead interface. Error bars represent SEM for 4–8 independent experiments of 50 cells each.

Rafts have been implicated in T cell polarity [48–50], so we tested the effect of mutating the raft-association motif on MTOC reorientation to the CD3/CD28 beads. MD45 cells expressing wild-type or mutated CD46 were treated with CD46 or control antibody prior to incubation with CD3/CD28 beads, and scored for MTOC polarisation to the beads. Transfection of the CD46 variants alone had no effect on MTOC polarisation (Figures 4(a) and 4(b), black bars, between 72% and 79% polarisation), but ligation of CD46Cyt1 reduced MTOC recruitment to the CD3/CD28 beads (Figure 4(a), 79% to 23%, *P* < .001). This effect was specific for the CD46 antibody, because antibodies to Tfr had no effect, and was mediated by CD46Cyt1 but not by CD46Cyt2 (Figure 4(a)). These data indicate that the competition for polarity between CD46 and TCR signals can be observed in MD45 cells expressing the Cyt1 isoform. In contrast, ligation of CD46Cyt1.C-A in which the raft-binding sites were mutated caused some inhibition of T-cell reorientation to the TCR signal (Figure 4(b), to 54%) but was not as effective as ligation of wild-type CD46Cyt1 (*P* < .001 for mutated CD46 compared with wild-type). The observation that reorientation was not completely abolished by mutating the raft-association site, and that CD46Cyt2 was not able to mediate reorientation, indicates that entities other than lipid rafts also play a role. Given that CD46 associates with the polarity regulator, Discs large (Dlg) via its C-terminus ([28] and see below), we tested whether mutation of the C-terminus could impact upon repolarisation. Indeed, mutation of the C-terminal residue (CD46.Cyt1L-R) significantly reduced the reorientation of the T cells, and this was even further reduced (but not completely abrogated) by a double mutation (CD46.Cty1C-A,L-R). These observations combined indicate that the association of CD46 with lipid rafts is necessary, but not sufficient, for the prevention by CD46 ligation of both raft and MTOC accumulation at TCR signalling sites, and suggest that association of CD46 with Dlg might also play a role.

## 4. Discussion

This study shows that the competing polarizing signal triggered by CD46 ligation [25] is controlled in part by the association of CD46 with lipid rafts. We correlate biochemical, microscopic, and functional experiments to indicate the following model. Firstly, CD46 is not constitutively associated with DRM but is located in domains that are slightly more ordered than the average membrane. We observed an increase in ordered domains on transfection of wild-type CD46-Cyt1 but not CD46-Cyt1C-A, suggesting the possibility that CD46 constitutively associates with a subset of small, unstable rafts. Secondly, ligation of CD46 causes partial recruitment into DRM and ordered membrane domains (and results in an increase in abundance of ordered domains), indicating that ligation causes membrane reorganisation and stabilization of raft domains. The palmitoylated transmembrane cysteine is critical for this recruitment, as measured both by Laurdan staining and by detergent extraction. Thirdly, CD46-mediated reorganisation of ordered membranes is partially responsible for the inhibition of T-cell polarization in response to TCR signals. 

Our observations provide a mechanism for our previous findings that competition for polarity by CD46 can profoundly affect the ability of T cells and NK cells to respond to activating signals and to mediate effector functions [25]. We find that the competition with TCR-induced polarity is mediated by CD46Cyt1, but not CD46Cyt2. This parallels an inhibitory effect of ligation of CD46Cyt1, but not CD46Cyt2, on CD8 effector function in vivo [24], suggesting that competition for polarity might play an important role in these in vivo effects. In this study we ligate CD46 using antibodies, but our previous work indicates that the measles hemagglutinin can exert a similar competition for polarity [25], and recruitment of CD46 into DRM by HHV6 infection [41] suggests that this pathogen might also reorient polarity. Similarities in signaling outcomes triggered by antibody and complement components [23] suggest that complement might also mediate a competition for polarity. Use of the CD46 mutations described herein will elucidate how broad a role competition for polarity might play in signaling through CD46, and whether this phenomenon is important in therapeutic applications of CD46-binding pathogens.

Interestingly, although these data indicate that recruitment into rafts is necessary for CD46-mediated effects on T-cell polarity, our data with CD46. Cyt2 indicates that recruitment of CD46 into rafts (as measured by both DRM extraction and Laurdan staining) is not sufficient to prevent polarisation of either rafts or the MTOC to the site of TCR stimulation. This, combined with our observation that abrogation of raft accumulation only partially prevents re-orientation, indicates that the dominant effect of CD46 on T-cell polarity is mediated by a combined contribution from lipid rafts and other entities. We have previously found that CD46 interacts with Dlg, a member of a network that regulates polarity of epithelial cells and T cells [27, 28, 48], making it a likely candidate to contribute to the re-orientation mediated by CD46. The reduced re-orientation of a mutated CD46.Cyt1 with abrogated binding to Dlg is compatible with the notion that CD46 interacts with the polarity network to mediate re-orientation. Further experimentation is required to confirm a role for Dlg, and to determine whether the slight defect in repolarisation of the double-mutant indicates that other entities are also involved.

## 5. Conclusion

These experiments indicate that CD46 associates with lipid rafts, and that this association facilitates the repolarisation of T cells triggered by CD46 ligation. CD46 ligation prevents the recruitment of rafts (this study) and the MTOC (this study and [25]) to the site of TCR stimulation, thus abrogating TCR signalling events [25]. That both raft and MTOC mis-orientation involves the transmembrane cysteine of CD46 implicates rafts as a central player in the competition for control of T cell polarity by receptor signalling. However, our observations that mutation of the cysteine does not completely abrogate CD46-mediated repolarisation, and that mutation of the Dlg-binding site can contribute to this effect, indicates that rafts act in cooperation with other morphological determinants. An important conclusion from these experiments is that the regulation of polarity cannot be explained by a hierarchical, stepwise contribution of the different components, but rather by a consensus decision based upon contributions from multiple components.

##  Authors' Contribution

K. Gaus and S. M. Russell contributed equally to the paper.

## Abbreviations

Tfr: Transferrin receptor
DRM: Detergent resistant membranes
TTX-100: Triton X-100
IS: Immunological synapse
HHV-6: Herpes virus 6.
